# Association between breast cancer and thyroid cancer risk: a two-sample Mendelian randomization study

**DOI:** 10.3389/fendo.2023.1138149

**Published:** 2023-05-23

**Authors:** Hong Tan, Sisi Wang, Feifei Huang, Zhongyi Tong

**Affiliations:** ^1^ Department of Pathology, The Second Xiangya Hospital, Central South University, Changsha, Hunan, China; ^2^ National Clinical Research Center for Metabolic Diseases, The Second Xiangya Hospital, Central South University, Changsha, Hunan, China; ^3^ Department of Medical Laboratory, Brain Hospital of Hunan Province (The Second People’s Hospital of Hunan Province), Changsha, Hunan, China; ^4^ Department of Pathology, Shenzhen People’s Hospital, Second Clinical Medical College of Jinan University, Shenzhen, Guangdong, China

**Keywords:** Mendelian randomization, breast cancer, thyroid cancer, causal effect, single nucleotide polymorphism

## Abstract

**Background:**

Breast and thyroid cancer are increasingly prevalent, but it remains unclear whether the observed associations are due to heightened medical surveillance or intrinsic etiological factors. Observational studies are vulnerable to residual confounding, reverse causality, and bias, which can compromise causal inference. In this study, we employed a two-sample Mendelian randomization (MR) analysis to establish a causal link between breast cancer and heightened thyroid cancer risk.

**Methods:**

We obtained the single nucleotide polymorphisms (SNPs) associated with breast cancer from a genome-wide association study (GWAS) conducted by the Breast Cancer Association Consortium (BCAC). The FinnGen consortium’s latest and largest accessible GWAS thyroid cancer data at the summary level. We performed four MR analyses, including the inverse-variance-weighted (IVW), weighted median, MR-Egger regression, and weighted mode, to evaluate the potential causal connection between genetically predicted breast cancer and higher risk for thyroid cancer. Sensitivity analysis, heterogeneity and pleiotropy tests were used to ensure the reliability of our findings.

**Results:**

Our study revealed causal relationship between genetically predicted breast cancer and thyroid cancer (IVW method, odds ratio (OR) = 1.135, 95% confidence interval (CI): 1.006 to 1.279, *P* = 0.038). However, there was no causal association between genetically predicted triple-negative breast cancer and thyroid cancer (OR = 0.817, 95% CI: 0.610 to 1.095, *P* = 0.177). There was no directional pleiotropy or horizontal pleiotropy in the present study.

**Conclusion:**

This two-sample MR study supports a causal link between ER-positive breast cancer and heightened the risk of thyroid cancer. Our analysis did not reveal a direct correlation between triple-negative breast cancer and thyroid cancer.

## Introduction

According to the American Cancer Society, breast cancer had the highest incidence (30%) of new cases and the second highest mortality rate (15%) among female deaths in the United States in 2020 (1). Globally breast cancer, accounted for 15% of all female cancer deaths, causing the deaths of 685,000 women in the same year (2, 3) The majority of breast cancer deaths resulted from recurrent or distant tumor metastases (4). Breast cancer can be categorized as either ER-positive or ER-negative depending on whether estrogen receptor (ER) expression is present on cancer cells. ​Approximately 70% of breast cancer patients express ER, making it a crucial therapeutic target. The most effective cancer treatment targets ER alpha activity in ER-positive breast cancer (5). When investigating the correlation between breast cancer and other diseases, it is important to consider the prognostic and predictive value of ER status as a stratification factor (6). Studies have suggested that breast cancer survivors are more likely to develop thyroid cancer, and thyroid cancer survivors are more likely to develop breast cancer (7, 8). The correlation between breast and thyroid cancer is thought to result from shared hormonal risk variables, detection bias, treatment effects, genetic vulnerability, cross-reactivity with autoantibodies (9), and hormonal crosstalk (10, 11). Clinicians should be especially aware of this correlation as the number of breast and thyroid cancer survivors continues to rise (8, 12).

So far, the epidemiological and clinical trial investigations have not established a causal relationship between breast cancer and thyroid cancer (13, 14). A retrospective case-control study that compared the genetic profiles of hereditary cancer risk genes between breast cancer and co-occurring breast-thyroid cancer patients found a genetic tendency for co-occurrence (15). Additionally, the risk of papillary thyroid cancer was found to be increased 1.3 times after the occurrence of breast cancer than in the general population. However, it was unclear whether the results were due to a common etiology or treatment effects. Recent studies have examined the link between breast cancer and thyroid cancer risk, but the results have been inconclusive. Consequently, there is a need for further investigation into the potential links between breast cancer and thyroid cancer.

In a study utilizing the Surveillance, Epidemiology and End Results (SEER) program cancer registries, another investigation was conducted into the potential association between breast cancer and thyroid cancer (16). However, the study was unable to fully exclude the possibility of a common etiology or the effects of treatment. In recent years, several studies have examined the risk of breast cancer in relation to thyroid cancer (17–19). The latest study on patients with differentiated thyroid cancer in children and young adults demonstrated a heightened risk of solid malignancies associated with radioactive iodine (RAI) treatment, with the risk of RAI-associated breast cancer being the most prominent after a 20-year follow-up (18). Conversely, a comprehensive analysis of the medical records of 4.24 million women did not identify a correlation between breast cancer survivors and the risk of thyroid cancer (19). Similarly, an earlier analysis involving ten thyroid cancer registries worldwide, including 6,449 patients treated with radioiodine for differentiated thyroid carcinoma and 1,116 controls, failed to reveal a substantial increase in the risk of breast cancer following radioiodine treatment. Nevertheless, the proportion of individuals receiving radioiodine treatment at a young age and the insufficient sample size precluded the establishment of statistically valid finding (20). The presence of residual or unmeasured confounding variables may also make it difficult to infer causal relationships from these results. As such, these findings underscore the need for a thorough investigation of the potential associations between breast cancer and thyroid cancer. One recent MR study used GWAS data to analyze the causal links between thyroid function and breast and thyroid cancer, and the results suggested a genetic predisposition for thyroid dysfunction to be associated with breast cancer.

An important aspect of conducting MR analysis is to investigate the causal effects of genetic variants that are strongly associated with potential risk factors on exposure and outcomes, as evident from previous studies (21–24). MR analysis has been shown to be more robust to measurement errors, confounding, and reverse causation compared to standard multivariate regression methods (25). The genetic variants included in the GWAS included 122,977 comprised a large sample of European and Asian ancestry breast cancer patients and controls, enabling the identification of variants that are highly related to overall, ER-negative and ER-positive breast cancer (26). Utilizing these data in conjunction with MR techniques, great advances have been made in the analysis of risk factors for follower-associated breast cancer (27, 28). Recently, a MR study was conducted using GWAS data to investigate causal links between thyroid function and breast and thyroid cancer, revealing that a genetic predisposition for thyroid dysfunction to be associated with breast cancer (29). However, it remains unknown whether thyroid cancer increases the risk of breast cancer.

While prior clinical observations and relevant published studies suggest that thyroid cancer and breast cancer are associated (7, 8), no MR study has examined this association. Therefore, a two-sample MR analysis was conducted to investigate the causal relationship between breast cancer and thyroid cancer.

## Methods

### Study design and MR assumptions

**Figure 1:**
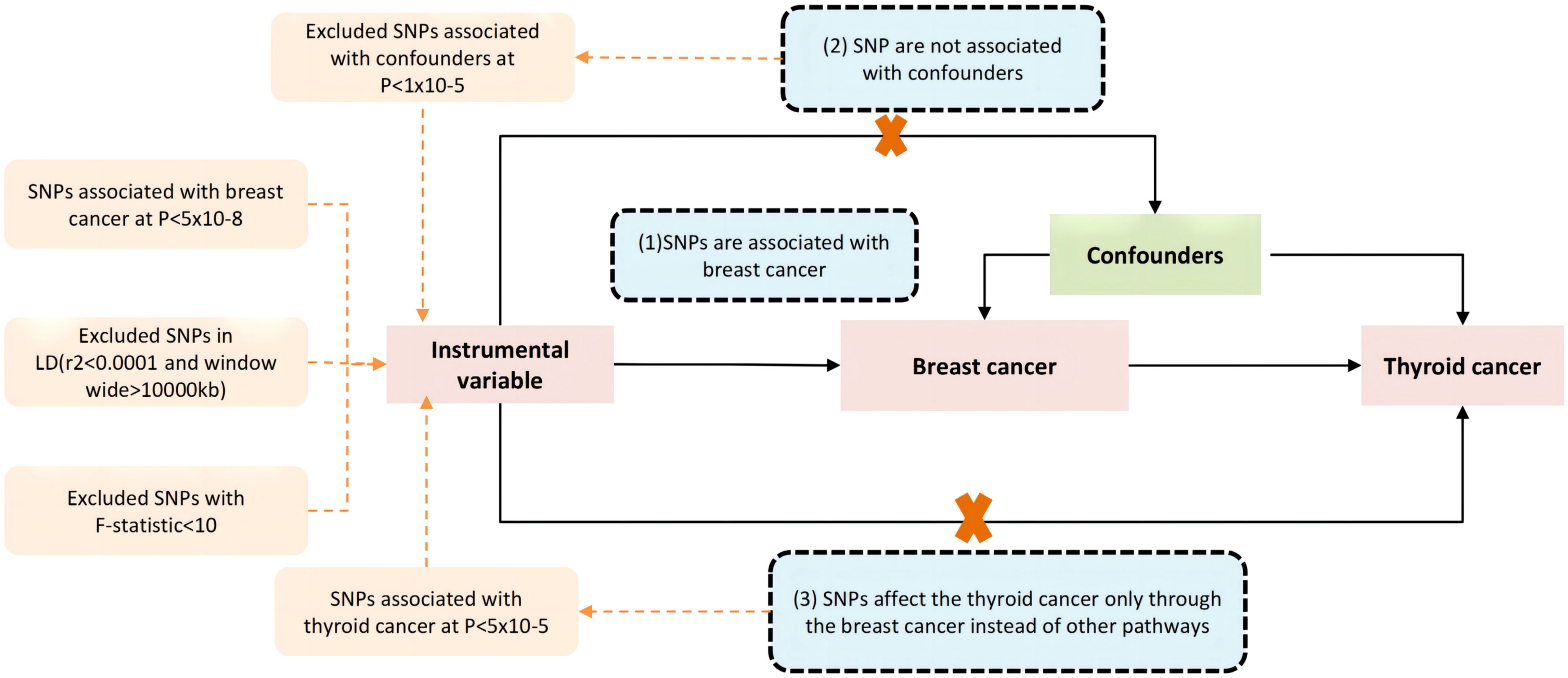
The core three instrumental variable assumptions. (1) the genetic variants have a strong association with the exposure(breast cancer), the threshold used for picking SNPs that are associated with breast cancer at genome wide significance (*P* < 5 × 10^−8^), and a threshold of r^2^ value of 0.001 and a 10,000 kb window used to excluded linkage disequilibrium (LD), (2) the genetic variants must not be associated with any other factors associated with the breast cancer and thyroid cancer connection, and (3) the genetic variants are not related with the thyroid cancer other than through breast cancer, by setting a threshold at genome wide significance (*P* < 5 × 10^−5^). Pathways that deviate from the assumptions are represented by the dotted line.

In our study, a two-sample Mendelian randomization (MR) design was employed to examine the causal relationship between breast cancer and thyroid cancer. To minimize bias resulting from data overlap, aggregated genetic data for breast cancer and thyroid cancer were obtained from the Breast Cancer Association Consortium (BCAC) (30) and the FinnGen research project (31). Our MR analysis was conducted based on three instrumental variable assumptions (32). Firstly, the relevance assumption was satisfied by selecting genetic variants that had a strong association with breast cancer at the genome-wide level of significance (*P*<5×10^−8^). Secondly, the independence assumption was ensured by verifying that the genetic variants were not associated with any other potential factors linked with breast cancer and thyroid cancer. Lastly, the exclusion-restriction assumption was met by setting a significance threshold at genome-wide (*P* > 5 × 10^−5^) and stipulating that the genetic variants were not related to thyroid cancer other than through breast cancer. A study frame diagram was provided in **Figure 1** to depict the design of our study.

### Study populations

The study obtained GWAS summary statistics from the Breast Cancer Association Consortium (BCAC) for a total of 133,384 cases with breast cancer and 113,789 breast cancer-free controls (30). The research project FinnGen was utilized to access the data of thyroid cancer patients. FinnGen offers researchers the opportunity to investigate the relationship between genetic variations and disease patterns in isolated populations. In the latest release 8 (R 8), Regeniehas several advantages, including fast leave-one-chromosome-out relatedness calculation to avoids proximal contamination, and utilization of an approximate Firth test which provides more reliable effect size estimates for rare variants. The total sample size for this release (R 8) was342,499 participants, consisting of 190,879 females and 151,620 males, and 20,175,454 variants were examined. Written informed consent was obtained from every participant, and the MVP was approved by the Veteran Affairs Central Institutional Review Board in accordance with the Helsinki Declaration.

### Breast cancer genetic data

In this study, we conducted an analysis of the Genome-Wide Association Study (GWAS) summary statistics from the Breast Cancer Association Consortium (BCAC) (30). The dataset included genetic information from a total of 133,384 breast cancer cases and 113,789 breast cancer-free controls. Moreover, we analyzed data specific to the triple-negative (TN) breast cancer risk stratum, which consisted of 18,016 patients and 100,974 controls. The updated data contained European ancestry women from 82 BCAC studies, involving more than 20 countries. The genetic data were obtained from the iCOGS array (26) and the OncoArray (33), and other GWAS data. The Haplotype Consortium (34) was used as a reference panel to help in the process of imputation of genotypes for variations that were not present on the arrays. Previous article (35) has provided specific descriptions of the procedures for imputation, sample quality control, and genotyping in detail. In our studies, we limited focused on SNPs with a minor allele frequency of at least 0.01 percent in at least one of the two datasets and were imputed with an imputation r^2^ of at least 0.7.

### Thyroid cancer genetic data

FinnGen research project is a public-private partnership endeavor that integrates genotype data from Finnish biobanks and digital health record data from the Finnish health registry. In this study, we conducted an analysis employing the data on malignant neoplasm of the thyroid gland with the exception of all cancers, encompassing a sample size of 1,525 cases and 259,583 controls,. It is noteworthy that this data was obtained from the FinnGen consortium’s latest release in December 2022 (31).

### Selection of genetic instruments

The breast cancer GWAS data were obtained from BCAC (30) at genome wide significance (*P* < 5 × 10^−8^). Then, we used linkage disequilibrium (LD) to assess the presence of gene linkage in these SNPS, and r^2^ value of 0.001 and 10,000 kb window. Secondly, to meet the independence assumption, we further screened Phenoscanner (http://www.phenoscanner.medschl.cam.ac.uk/) to determine the potential pleiotropic confounders. Then, those exposure-related SNPs were deleted from the GWAS of thyroid cancer. Third, to meet the third assumptions (genetic variants solely-influence the outcome through the risk-factor), we excluded those SNPs that directly related with the thyroid cancer by setting a threshold at genome-wide significance (*P* > 5 × 10^-5^). Additionally, we computed *F*-statistics (36) [ $F=\left(\frac{R^{2}}{1-R^{2}}\right)\left(\frac{n-k-1}{k}\right)$ , R^2 = ^2×(1-MAF)×MAF×β^2)^], and weak IVs (*F* < 10) were deleted.

### Confounders

There exist several factors that may act as potential confounders in the relationship between breast cancer and thyroid cancer. According to the most recent study (37), the risk factors identified included central obesity, diastolic blood pressure, HbA1c, and telomere length. To verify the association of these risk factors with SNPs, we utilized the PhenoScanner (accessible at http://www.phenoscanner.medschl.cam.ac.uk/) and confirmed the inclusion of SNPs in our analysis (38). Our examination revealed that four SNPs were related with the potential risk factors of thyroid cancer: rs1432679 was associated with diastolic blood pressure, while rs2588808, rs55872725, and rs78440108 were linked to central obesity. Then, to satisfy MR’s premise that genetic variation should not be associated with confounding factors in expose-outcome relationships, we eliminated those 4 SNPs from the SNP dataset.

### Statistical methods

We performed two sample MR analyses to investigate the association between breast and thyroid cancers risk and genetic instruments. Specifically, we analyzed breast cancer risk using data from a large-scale GWAS of BCAC (sample 1). The latest data from the FinGen Consortium (sample 2) was used to estimate of an association between a genetic instrument and thyroid cancer. Four alternative approaches to MR [random-effect inverse-variance weighted (IVW), weighted mode and MR Egger, and weighted median] were applied to determine the potential influence of variation heterogeneity and pleiotropy (21). As a primary MR method, the IVW method produces unbiased estimations when horizontal pleiotropy does not exist or is balanced. The other three methods were used to analyze causal estimates even in the presence of unbalanced pleiotropy (39).We evaluated the presence of horizontal pleiotropy through the MR-Egger intercept test (40). In addition, a leave-one-out analysis was carried out in order to evaluate whether or not a single SNP was responsible for a significant effect. Furthermore, the Cochran’s Q test was alsp utilized in order to identify any instances of heterogeneity. Funnel plot was used to evaluate the probable directionality of pleiotropy (41).

All analyses were performed based on TwoSampleMR (version 0.5.6) and R software (version 4.2.1). The level of significance for the test was established at 0.05 for the *P*-value (two-sided).

## Results

### Selection of instrumental variables

For the instrumental variable of breast cancer, at first there are 8063 SNPs reached the threshold at genome wide significance level (*P* < 5 × 10^−8^), following the matching with the GWAS of thyroid cancer, only 65 SNPs remained. Furthermore, after assessing the SNP dataset using in PhenoScanner (38), 4 SNPs were removed due to their association with confounding variables. Consequently, 61 SNPs selected for evaluating the genetic risk of thyroid cancer in patients breast cancer. Notably, all the *F*- statistics range from 318.05 to 10462.41, indicating the presence of strong instruments (36).

Regarding the instrumental variables of TN breast cancer, a total of 2018 SNPs initially reached the genome-wide significance threshold (*P* < 5 × 10^−8^). After matching with the GWAS of thyroid cancer, only 25 SNPs remained. Among these, 4 SNPs (rs12472404, rs2735846, rs6585204, and rs67397200) were discarded due to their palindromic and ambiguous structure. Ultimately, 19 SNPs were selected for assessing the genetic risk of thyroid cancer in patients with TN breast cancer, and all *F*-statistics ranged from 250.29 to 1122.63, indicating the presence of strong instruments (36). Additional details on the SNPs are presented in **Supplement Tables 1, 2**.

### MR analysis of breast cancer with risk of thyroid cancer

**Figure 2:**
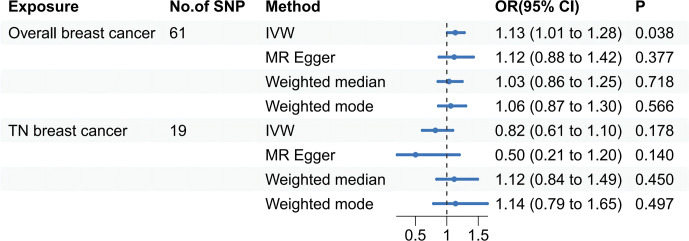
A forest plot shows the odds ratios (ORs) and 95% confidence intervals (CIs) for the effect of breast cancer on thyroid cancer, and triple-negative breast cancer on thyroid cancer using the two-sample Mendelian randomization. IVW stands for inverse variance weighted. TN breast cancer stands for triple-negative breast cancer.

**Figure 3:**
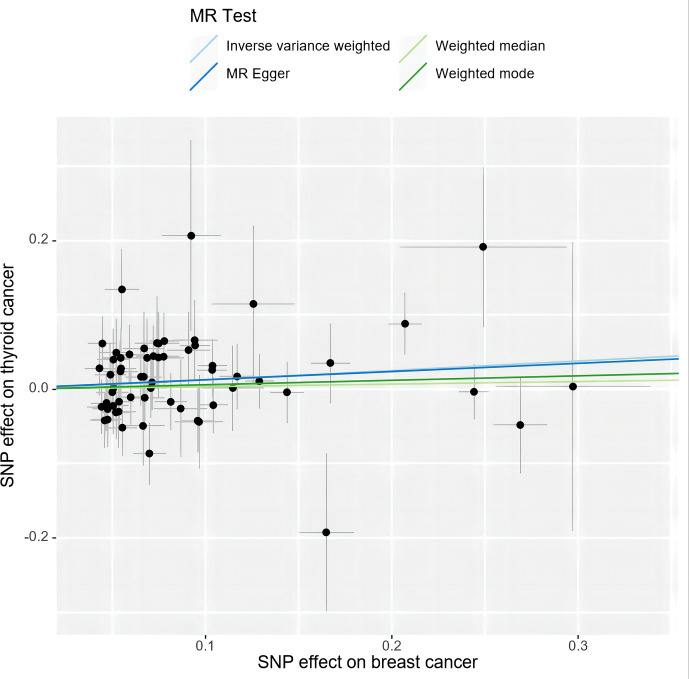
A scatter plot shows the effects of SNPs on breast cancer and thyroid cancer. MR stands for Mendelian randomization. IVW, MR-Egger, weighted median, and weighted mode slopes represent results from these regression analyses.

**Figure 4:**
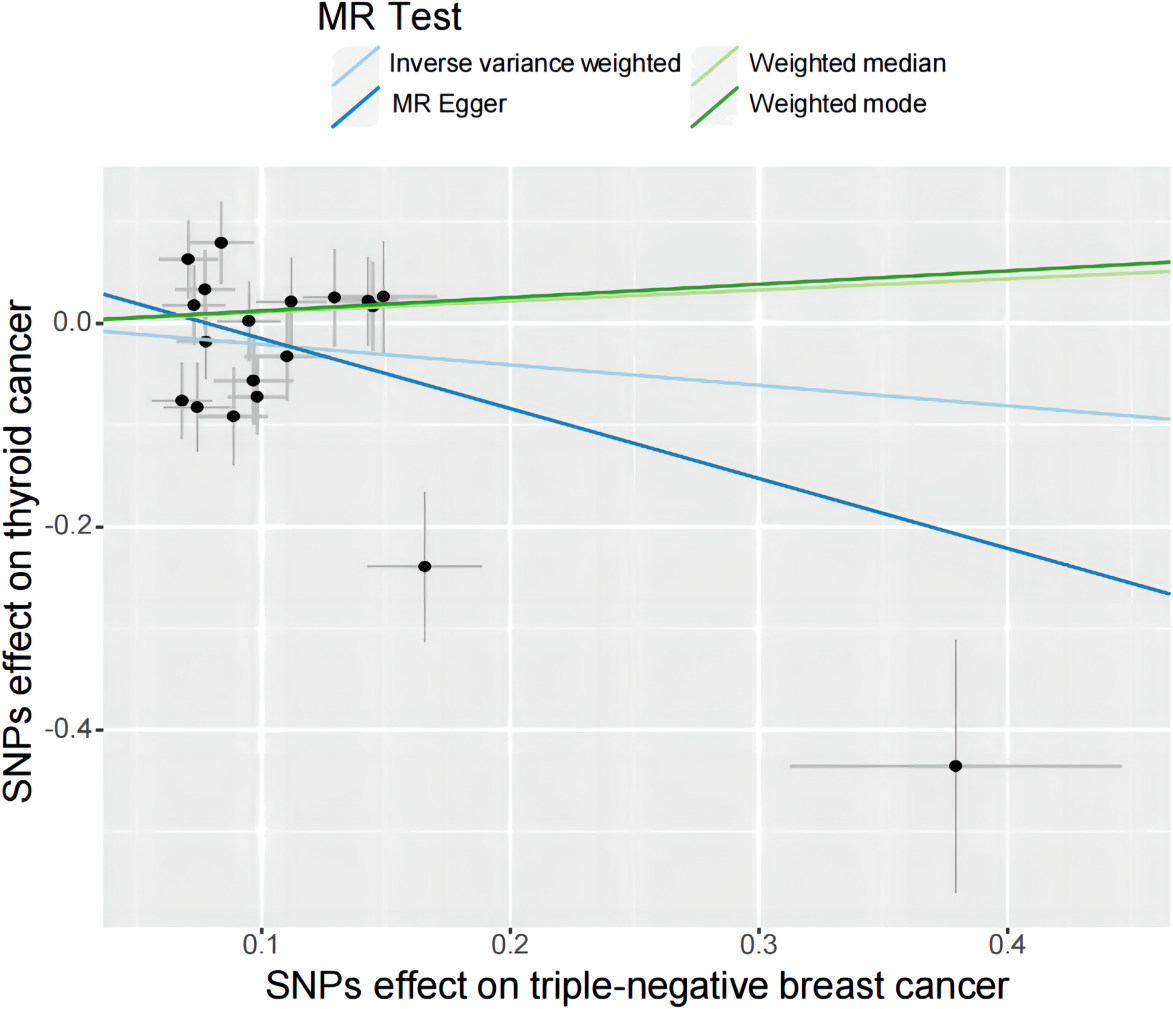
A scatter plot shows the effects of SNPs on triple-negative breast cancer and thyroid cancer. MR stands for Mendelian randomization. IVW, MR-Egger, weighted median, and weighted mode slopes represent results from these regression analyses.

Specifically, results of the IVW method revealed significantly elevated risk of thyroid cancer in individuals with breast cancer (Odds ratio (OR) =1.135, 95% CI: 1.006 to 1.279, *P* = 0.038 per genetically predicted one-SD increase), whereas MR-Egger, weighted median method, and weighted mode produced more conservative estimates that did not attain statistical significance (MR-Egger: OR=1.116, 95% CI: 0.875 to1.424, *P*=0.377; weighted median: OR=1.034, 95% CI: 0.858 to1.247, *P*=0.718; weighted mode: OR=1.062, 95% CI: 0.865 to 1.302, *P*= 0.566) (**Figure 2**). The study findings indicate that the estimated causal effects of total breast cancer were highly consistent across IVW, median weighted, and weighted modes for total breast cancer, despite the fact that the significance differed across approaches (**Figure 3**). However, there was no evidence of a causal link between TN breast cancer and thyroid cancer (by the IVW method, OR= 0.817, 95% CI: 0.610 to 1.095; *P* = 0.177) (**Figure 2**). The estimated causal effects of TN breast cancer and thyroid cancer were unconsistent across IVW, median weighted, and weighted modes for total breast cancer, indicated no causal link between TN breast cancer and thyroid cancer (**Figure 4**).

**Figure 5:**
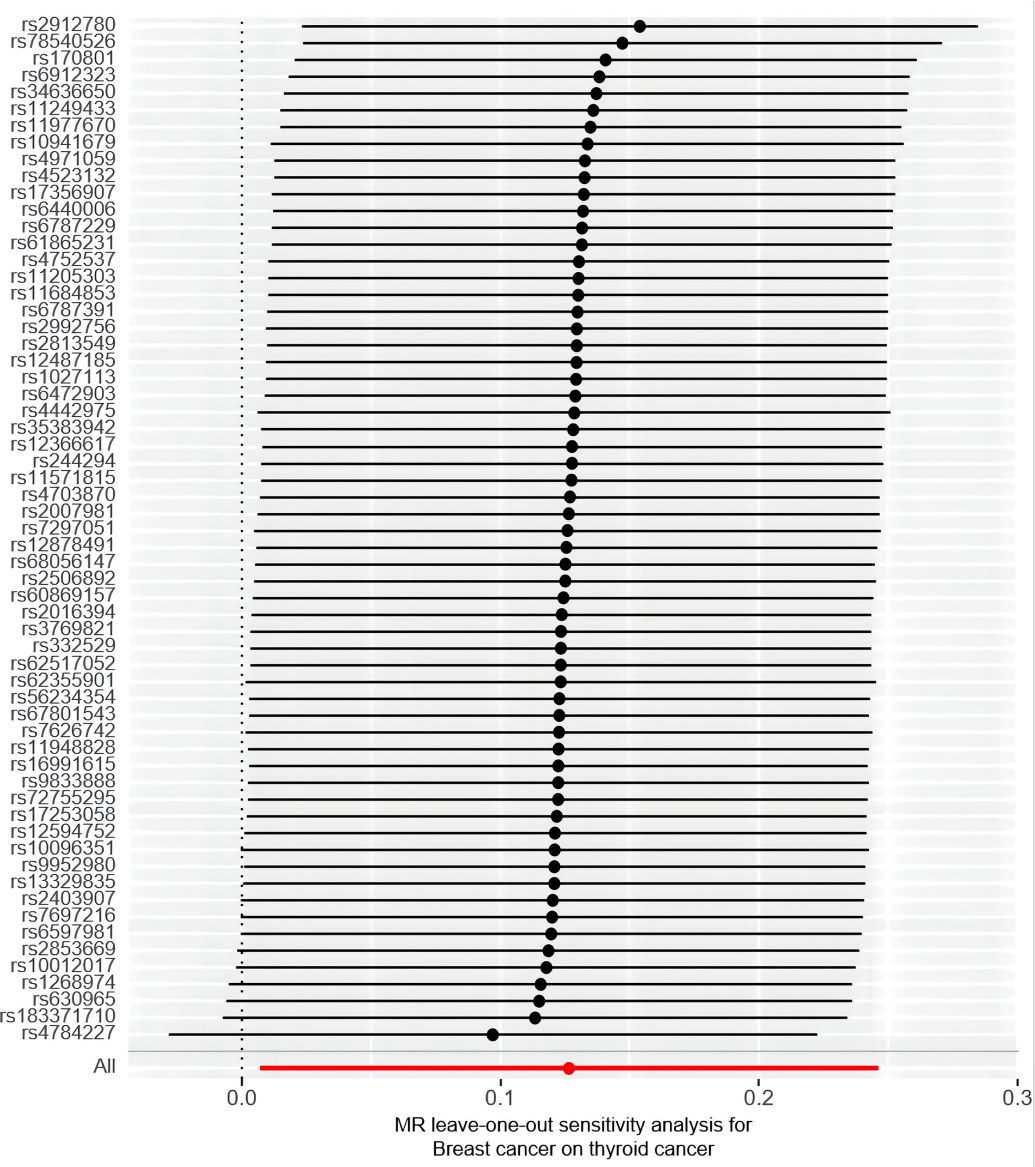
A leave-one-out analysis of the estimations for breast cancer and thyroid cancer.

Furthermore, the sensitivity analyses performed in this study support robustness of the observed causal estimates, as all MR sensitivity analyses yielded significant results, and the MR-Egger regression test’s intercept indicated no significant directed pleiotropy in the overall breast cancer population (intercept = 0.011, *P* = 0.883). Additionally, the leave-one-out or single SNP analysis showed no evidence of significant disproportionate effect for any given SNP on the causal estimates (**Figure 5**). The results showed that there was heterogeneity among SNPs (Cochran’s Q value = 58.103, *P* = 0.545). The funnel plot also indicated the absence of horizontal pleiotropy, as the causal effect of variables corresponded to their precision (**Supplement Figure S1**).

**Figure 6:**
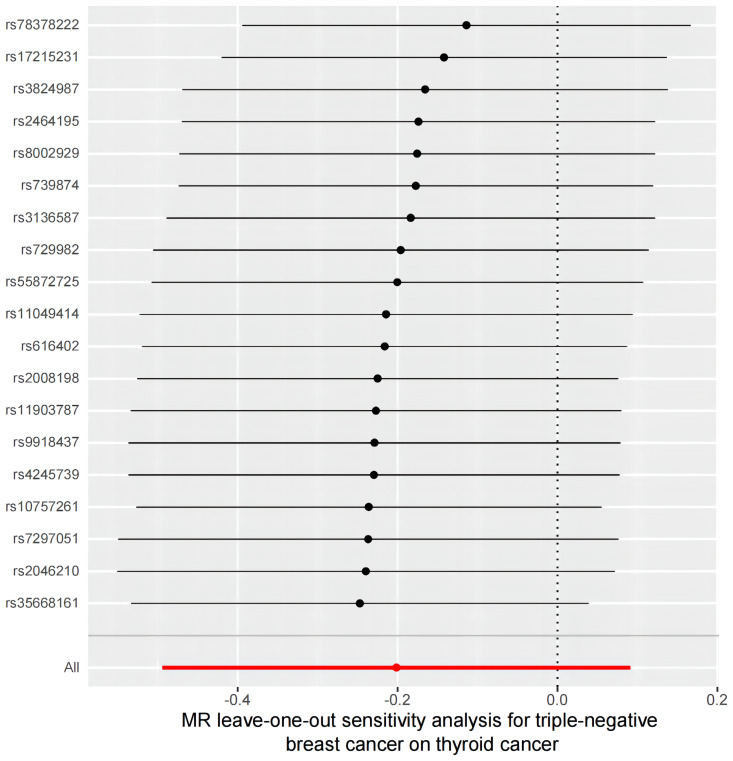
A leave-one-out analysis of the estimations for triple-negative breast cancer and thyroid cancer.

However, the sensitivity analyses for TN breast cancer and thyroid cancer showed heterogeneity (Cochran’s Q value = 43.72, *P* < 0.001). Nonetheless, there was no significant directional pleiotropy in the overall breast cancer population (intercept = 0.054, *P* = 0.261), and the leave-one-out analysis indicated no significant disproportionate effect for any given SNP on the causal estimates (**Figure 6**). Funnel plots, where the causal effect of an estimated variable corresponds to its accuracy, also indicated the absence of horizontal pleiotropy (**Supplement Figure S2**).

## Discussion

MR is a method that uses genetic diversity to study potential causal relationships between risk factors and health outcomes. Using large-scale genetic data sets and two-sample MR analysis, we provide evidence that higher genetic susceptibility to breast cancer is associated with a greater risk of overall thyroid cancer, but this association is not present in TN breast cancer.

Breast and thyroid cancers are prevalent tumors in women, and they often co-occur (42). There was a study by Goldgar et al. studied the risk of cancer among first-degree relatives of cancer probands, and they found that first-degree relatives of breast cancer patients had an increased risk of thyroid cancer (43). Previous research has identified probable causal factors in both cancers, such as the thyroid gland, estrogen signaling, lifestyle, and environmental factors (14). However, the causal link between breast cancer and thyroid cancer remains uncertain, and new preventative measures, diagnostic tests, and therapies cannot be developed until the underlying causes of the secondary primary tumors are understood (44). Our primary investigation, which included genetic tests at a significant level of *P* < 5 x 10^-8^, yielded compelling evidence supporting the hypothesis that higher genetic susceptibility to breast cancer is associated with a greater risk of thyroid cancer. Another related study (8) showed that thyroid cancer is more likely to occur as a secondary malignancy after breast cancer, and following thyroid cancer, there is a greater risk of breast cancer as a secondary cancer. There was a reduced incidence of breast cancer in 134,122 women with hypothyroidism who participated in the study by Chien-Hsiang Weng (17). A two-sample MR study found a causal relationship between thyroid dysfunction and an increased risk of breast cancer (specifically ER-positive tumors) (29). Our study fills a gap in current research by analyzing the causal association between thyroid cancer and breast cancer. Earlier research suggests that the co-morbidity of thyroid and breast malignancies may be due to immunologically significant cross-talk, as the expression of estrogen receptor (ER) and thyroid hormone receptor (TR) was observed to increase, potentially contributing to the co-morbidity (45). We found that thyroid peroxidase, which is widely expressed in both the thyroid and breast, could be the key antigenic connection between thyroid autoimmunity and breast cancer. It also reacts with certain autoantibodies that target thyroid peroxidase (9). However, other researches have suggested that radiation therapy following cancer treatment may influence this causal association, but recent studies have not found a significant link. Using Cancer Registry and SEER data, two studies found that radiation therapy for breast cancer did not significantly increase the incidence of thyroid cancer (46, 47). In fact, the eventual incidence of thyroid cancer was consistently higher in groups that did not get radiation therapy, which suggested that radiotherapy may not be a major risk factor, and that the causal relationship between these two cancers is worth investigating.

Our work using comprehensive genetic data from 342,499 individuals in GWAS provides evidence for a causal link between breast cancer and thyroid cancer in ER-positive breast cancer but not in triple negative breast cancer. We accounted for all breast cancer features and the cancer risk stratum (ER status) to arrive at a causal effect estimate. While our findings do not provide proof of a particular mechanism for carcinogenesis, they highlight possible pathways that require further investigation.

However, our study has several limitations. We used summary-level data and subgroup analyses by ER status, but we could not stratify breast cancer analyses by other cancer risk variables (e.g. BMI, exogenous hormone use). MR can only draw conclusions regarding trait connections in the populations from which the GWAS are obtained. Finally, although our study group is of European descent, the European community is ethnically diverse, and our study cohort may not be fully representative.

## Conclusion

The results of this MR analysis provide evidence in support of a causal link between an elevated ER-positive breast cancer risk and an increased risk of thyroid cancer. Further investigations into this relationship are warranted in the future. However, our data did not reveal a direct association between TN breast cancer and thyroid cancer, indicating that the preventative and control measures employed to address post-thyroid changes may not confer benefits for individuals with TN breast cancer. This study highlights the effectiveness of this analytical technique in identifying causal relationships, which may facilitate the identification of additional cancer-related links in the future.

## Data availability statement

The datasets presented in this study can be found in online repositories. The names of the repository/repositories and accession number(s) can be found in the article/**Supplementary Material**.

## Ethics statement

The studies involving human participants were reviewed and approved by FinnGen research project. The patients/participants provided their written informed consent to participate in this study.

## Author contributions

HT and ZT conducted analyses and wrote the first draft of the paper. ZT and HT acquired the funding. FH and SW contributed to data verification. All authors contributed to the article and approved the submitted version.
