# Supplementary material for: Association between breast cancer and thyroid cancer risk: a two-sample Mendelian randomization study
Source: Front Endocrinol (Lausanne). 2023 May 23;14:1138149. doi: 10.3389/fendo.2023.1138149 (PMC10242035; doi:10.3389/fendo.2023.1138149)
Supplement: Supplementary file 1 [file DataSheet_1.docx]

Table S1 The information of overall breast cancer SNPs

| SNP | effect allele | other allele | β | MAF | se | pval | R2 | F |
| --- | --- | --- | --- | --- | --- | --- | --- | --- |
| rs10012017 | T | G | 0.055293 | 0.256709 | 0.009224 | 2.04E-09 | 0.001167 | 399.8274 |
| rs10096351 | G | A | 0.10384 | 0.573188 | 0.007946 | 4.98E-39 | 0.005276 | 1815.488 |
| rs1027113 | A | G | 0.086753 | 0.919367 | 0.014209 | 1.02E-09 | 0.001116 | 382.3769 |
| rs10941679 | G | A | 0.143762 | 0.273067 | 0.009072 | 1.47E-56 | 0.008205 | 2831.803 |
| rs11205303 | C | T | 0.047544 | 0.411694 | 0.008039 | 3.33E-09 | 0.001095 | 375.2195 |
| rs11249433 | G | A | 0.104255 | 0.433067 | 0.008001 | 8.23E-39 | 0.005337 | 1836.698 |
| rs11571815 | A | G | 0.297191 | 0.010696 | 0.041572 | 8.75E-13 | 0.001869 | 641.003 |
| rs11684853 | T | G | -0.04434 | 0.545598 | 0.007934 | 2.29E-08 | 0.000975 | 334.0444 |
| rs11948828 | A | C | 0.05225 | 0.25115 | 0.009217 | 1.44E-08 | 0.001027 | 351.8654 |
| rs11977670 | A | G | 0.055486 | 0.446184 | 0.00796 | 3.16E-12 | 0.001521 | 521.5962 |
| rs12366617 | A | C | -0.05009 | 0.284456 | 0.008681 | 7.89E-09 | 0.001022 | 350.034 |
| rs12487185 | G | A | 0.046989 | 0.319642 | 0.008505 | 3.29E-08 | 0.00096 | 329.0365 |
| rs12594752 | T | C | -0.09096 | 0.124605 | 0.011705 | 7.79E-15 | 0.001805 | 618.97 |
| rs1268974 | G | A | -0.07799 | 0.59625 | 0.008076 | 4.57E-22 | 0.002929 | 1005.47 |
| rs12878491 | A | C | -0.06697 | 0.202209 | 0.009704 | 5.16E-12 | 0.001447 | 496.0378 |
| rs13329835 | G | A | 0.072201 | 0.240655 | 0.009349 | 1.14E-14 | 0.001905 | 653.4003 |
| rs16991615 | A | G | 0.092433 | 0.068193 | 0.016012 | 7.80E-09 | 0.001086 | 372.0681 |
| rs170801 | A | C | -0.06998 | 0.261691 | 0.008874 | 3.13E-15 | 0.001892 | 648.938 |
| rs17253058 | A | G | 0.067207 | 0.152936 | 0.01117 | 1.78E-09 | 0.00117 | 401.0482 |
| rs17356907 | G | A | -0.08143 | 0.279588 | 0.008677 | 6.33E-21 | 0.002671 | 916.7756 |
| rs183371710 | T | C | 0.24898 | 0.009844 | 0.044903 | 2.94E-08 | 0.001208 | 414.1558 |
| rs2007981 | A | C | -0.07142 | 0.323601 | 0.008373 | 1.48E-17 | 0.002233 | 765.9736 |
| rs2016394 | A | G | -0.04322 | 0.460915 | 0.007916 | 4.76E-08 | 0.000928 | 318.0564 |
| rs2403907 | A | C | -0.07763 | 0.300845 | 0.008551 | 1.10E-19 | 0.002535 | 870.0867 |
| rs244294 | G | A | -0.07076 | 0.288106 | 0.00865 | 2.83E-16 | 0.002054 | 704.4072 |
| rs2506892 | A | G | -0.06553 | 0.304193 | 0.008504 | 1.30E-14 | 0.001818 | 623.3048 |
| rs2813549 | T | C | 0.053482 | 0.769747 | 0.009301 | 8.93E-09 | 0.001014 | 347.4151 |
| rs2853669 | G | A | -0.07498 | 0.294277 | 0.008563 | 2.02E-18 | 0.002335 | 801.2653 |
| rs2912780 | T | C | -0.24409 | 0.533242 | 0.008016 | 1.00E-200 | 0.029659 | 10462.41 |
| rs2992756 | C | T | -0.05995 | 0.499066 | 0.007875 | 2.69E-14 | 0.001797 | 616.1577 |
| rs332529 | A | G | -0.06878 | 0.149279 | 0.010936 | 3.18E-10 | 0.001202 | 411.8054 |
| rs34636650 | A | G | -0.09587 | 0.2438 | 0.009059 | 3.56E-26 | 0.003389 | 1163.942 |
| rs35383942 | T | C | 0.114758 | 0.062571 | 0.016757 | 7.47E-12 | 0.001545 | 529.6415 |
| rs3769821 | T | C | -0.05477 | 0.650983 | 0.008323 | 4.68E-11 | 0.001363 | 467.2228 |
| rs4442975 | T | G | -0.12893 | 0.465398 | 0.00785 | 1.29E-60 | 0.008272 | 2854.938 |
| rs4523132 | T | C | -0.046 | 0.410036 | 0.008026 | 9.95E-09 | 0.001024 | 350.7698 |
| rs4703870 | A | G | 0.050714 | 0.757267 | 0.009133 | 2.81E-08 | 0.000945 | 323.9471 |
| rs4752537 | G | A | -0.05052 | 0.317399 | 0.008476 | 2.52E-09 | 0.001106 | 378.9815 |
| rs4784227 | T | C | 0.207132 | 0.285011 | 0.009009 | 5.61E-117 | 0.017486 | 6091.856 |
| rs4971059 | A | G | 0.047553 | 0.358665 | 0.008243 | 7.98E-09 | 0.00104 | 356.4589 |
| rs56234354 | A | G | 0.050665 | 0.277998 | 0.008838 | 9.90E-09 | 0.00103 | 353.0832 |
| rs60869157 | C | T | 0.054477 | 0.236406 | 0.009385 | 6.44E-09 | 0.001071 | 367.1508 |
| rs61865231 | A | G | 0.06655 | 0.136193 | 0.011626 | 1.04E-08 | 0.001042 | 357.0733 |
| rs62355901 | C | T | 0.167036 | 0.183489 | 0.010525 | 1.00E-56 | 0.00836 | 2885.835 |
| rs62517052 | C | T | 0.074856 | 0.102712 | 0.013579 | 3.54E-08 | 0.001033 | 353.9071 |
| rs630965 | T | C | 0.094517 | 0.645672 | 0.008184 | 7.52E-31 | 0.004088 | 1404.909 |
| rs6440006 | A | G | 0.052042 | 0.444012 | 0.007959 | 6.21E-11 | 0.001337 | 458.3413 |
| rs6472903 | T | G | 0.067464 | 0.839158 | 0.010561 | 1.68E-10 | 0.001229 | 421.0726 |
| rs6597981 | G | A | 0.044786 | 0.522198 | 0.007895 | 1.41E-08 | 0.001001 | 342.9493 |
| rs67801543 | T | C | 0.074061 | 0.128613 | 0.011989 | 6.52E-10 | 0.001229 | 421.346 |
| rs6787229 | A | G | -0.09662 | 0.096243 | 0.01316 | 2.11E-13 | 0.001624 | 556.7652 |
| rs6787391 | T | C | 0.05368 | 0.379314 | 0.008168 | 4.97E-11 | 0.001357 | 465.0685 |
| rs68056147 | A | G | 0.049089 | 0.306789 | 0.008592 | 1.11E-08 | 0.001025 | 351.1908 |
| rs6912323 | T | G | 0.164809 | 0.084549 | 0.014838 | 1.16E-28 | 0.004205 | 1445.336 |
| rs72755295 | G | A | 0.125799 | 0.034552 | 0.022257 | 1.59E-08 | 0.001056 | 361.7862 |
| rs7297051 | T | C | -0.11703 | 0.221441 | 0.00935 | 6.10E-36 | 0.004722 | 1624.036 |
| rs7626742 | T | G | 0.103684 | 0.56146 | 0.007956 | 8.08E-39 | 0.005294 | 1821.755 |
| rs7697216 | C | T | 0.094265 | 0.893385 | 0.012474 | 4.12E-14 | 0.001693 | 580.3991 |
| rs78540526 | T | C | 0.268911 | 0.093307 | 0.014531 | 1.84E-76 | 0.012235 | 4240.053 |
| rs9833888 | T | G | 0.054616 | 0.230284 | 0.009433 | 7.04E-09 | 0.001057 | 362.35 |
| rs9952980 | C | T | -0.05931 | 0.339757 | 0.008263 | 7.12E-13 | 0.001578 | 540.9999 |

Note: SNP: single-nucleotide polymorphism; MAF: minor allele frequency; SE: standard error; β: standard deviation change in breast cancer per copy of the effected allele.

Table S2 The information of triple-negative breast cancer SNPs

| SNP | effect_allele | other_allele | beta | eaf | se | pval | R2 | F |
| --- | --- | --- | --- | --- | --- | --- | --- | --- |
| rs10757261 | G | A | -0.0705 | 0.6304 | 0.012 | 4.58E-09 | 0.002316 | 276.2273 |
| rs11049414 | G | A | -0.0949 | 0.3085 | 0.0128 | 1.26E-13 | 0.003842 | 458.9706 |
| rs11903787 | A | G | -0.1119 | 0.2471 | 0.0141 | 1.82E-15 | 0.004659 | 556.9696 |
| rs17215231 | T | C | -0.1656 | 0.0792 | 0.0232 | 8.57E-13 | 0.004 | 477.8426 |
| rs2008198 | C | T | 0.0772 | 0.3732 | 0.0122 | 2.40E-10 | 0.002788 | 332.6986 |
| rs2046210 | A | G | 0.1428 | 0.3557 | 0.0121 | 5.57E-32 | 0.009347 | 1122.638 |
| rs2464195 | A | G | -0.068 | 0.3721 | 0.0122 | 2.48E-08 | 0.002161 | 257.6562 |
| rs3136587 | G | A | -0.0967 | 0.1613 | 0.0161 | 1.76E-09 | 0.00253 | 301.8056 |
| rs35668161 | A | C | 0.0838 | 0.2758 | 0.0131 | 1.56E-10 | 0.002805 | 334.7295 |
| rs3824987 | G | A | -0.0982 | 0.4333 | 0.0119 | 1.34E-16 | 0.004736 | 566.1867 |
| rs4245739 | A | C | -0.1293 | 0.7303 | 0.0129 | 1.51E-23 | 0.006586 | 788.8276 |
| rs55872725 | T | C | -0.0776 | 0.4169 | 0.0119 | 6.43E-11 | 0.002928 | 349.3855 |
| rs616402 | T | C | -0.0728 | 0.3049 | 0.0128 | 1.35E-08 | 0.002246 | 267.9029 |
| rs7297051 | T | C | -0.1448 | 0.2344 | 0.014 | 4.74E-25 | 0.007525 | 902.2157 |
| rs729982 | C | T | 0.1102 | 0.1269 | 0.0172 | 1.37E-10 | 0.002691 | 321.0645 |
| rs739874 | G | A | -0.0741 | 0.2574 | 0.0136 | 4.52E-08 | 0.002099 | 250.2912 |
| rs78378222 | G | T | -0.3791 | 0.0092 | 0.0668 | 1.36E-08 | 0.00262 | 312.5748 |
| rs8002929 | G | A | 0.0887 | 0.7582 | 0.0139 | 1.91E-10 | 0.002885 | 344.2509 |
| rs9918437 | T | G | 0.149 | 0.0728 | 0.0217 | 6.52E-12 | 0.002997 | 357.696 |

Note: SNP: single-nucleotide polymorphism; MAF: minor allele frequency; SE: standard error; β: standard deviation change in triple-negative breast cancer per copy of the effected allele.

1

2

3

4

5

6

-1

0

1

2

β

I

V

1

S

E

I

V

MR Method

Inverse variance weighted

MR Egger

Figure S1. Funnel plot for the overall breast cancer of SNPs. IVW stands for inverse variance weighted; MR stands for Mendelian randomization; IV, instrumental variable

2.0

2.5

3.0

-1.5

-1.0

-0.5

0.0

0.5

1.0

β

I

V

1

S

E

I

V

MR Method

Inverse variance weighted

MR Egger

Figure S2. Funnel plot for the triple-negative breast cancer of SNPs. IVW stands for inverse variance weighted; MR stands for Mendelian randomization; IV, instrumental variable

Data sources

The breast cancer genome-wide association analyses for BCAC and CIMBA were supported by Cancer Research UK (PPRPGM-Nov20\100002, C1287/A10118, C1287/A16563, C1287/A10710, C12292/A20861, C12292/A11174, C1281/A12014, C5047/A8384, C5047/A15007, C5047/A10692, C8197/A16565) and the Gray Foundation, The National Institutes of Health (CA128978, X01HG007492- the DRIVE consortium), the PERSPECTIVE project supported by the Government of Canada through Genome Canada and the Canadian Institutes of Health Research (grant GPH-129344) and the Ministère de l’Économie, Science et Innovation du Québec through Genome Québec and the PSRSIIRI-701 grant, the Quebec Breast Cancer Foundation, the European Community's Seventh Framework Programme under grant agreement no. 223175 (HEALTH-F2-2009-223175) (COGS), the European Union's Horizon 2020 Research and Innovation Programme (634935 and 633784), the Post-Cancer GWAS initiative (U19 CA148537, CA148065 and CA148112 - the GAME-ON initiative), the Department of Defence (W81XWH-10-1-0341), the Canadian Institutes of Health Research (CIHR) for the CIHR Team in Familial Risks of Breast Cancer (CRN-87521), the Komen Foundation for the Cure, the Breast Cancer Research Foundation and the Ovarian Cancer Research Fund. All studies and funders are listed in Zhang H et al (Nat Genet, 2020).
